# Targeted Metabolomic Approach to Assess the Reproducibility of Plasma Metabolites over a Four Month Period in a Free-Living Population

**DOI:** 10.1021/acs.jproteome.1c00440

**Published:** 2022-01-03

**Authors:** Xiaofei Yin, Orla Prendiville, Aoife E. McNamara, Lorraine Brennan

**Affiliations:** † UCD School of Agriculture and Food Science, Institute of Food and Health, University College Dublin, Belfield, Dublin 4 D4 V1W8, Ireland; ‡ UCD Conway Institute of Biomolecular and Biomedical Research, University College Dublin, Belfield, Dublin 4 D4 V1W8, Ireland

**Keywords:** reproducibility, plasma metabolites, targeted metabolomics, lipids

## Abstract

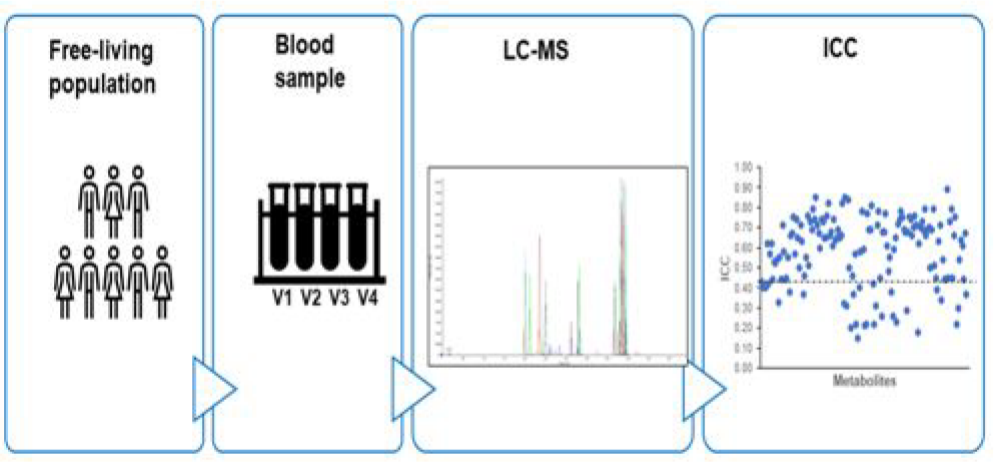

Metabolomics is increasingly applied to investigate diet–disease associations in nutrition research. However, studies of metabolite reproducibility are limited, which could hamper their use within epidemiologic studies. The objective of this study was to evaluate the metabolite reproducibility during 4 months in a free-living population. In the A-DIET Confirm study, fasting plasma and dietary data were collected once a month for 4 months. Metabolites were measured using liquid chromatography tandem mass spectrometry, and their reproducibility was estimated using the intraclass correlation coefficient (ICC). Regularized canonical correlation analysis (rCCA) was employed to examine the diet–metabolite associations. In total, 138 metabolites were measured, and median ICC values of 0.49 and 0.65 were found for amino acids and biogenic amines, respectively. Acylcarnitines, lysophosphatidylcholines, phosphatidylcholines, and sphingomyelins had median ICC values of 0.69, 0.66, 0.63, and 0.63, respectively. The median ICC for all metabolites was 0.62, and 54% of metabolites had ICC values ≥0.60. Additionally, the rCCA heat map revealed positive correlations between dairy/meat intake and specific lipids. In conclusion, more than half of the metabolites demonstrated good to excellent reproducibility. A single measurement per subject could appropriately reflect the metabolites’ long-term concentration levels and may also be sufficient for assessing disease risk in epidemiologic studies. The study data are deposited in MetaboLights (MTBLS3428 (www.ebi.ac.uk/metabolights)).

## Introduction

Metabolomics has considerable potential as an analytical tool to rapidly obtain information on the metabolic fingerprints/metabolites of individuals. These metabolites reflect the multiple influences of genetics, the microbiome, and environmental factors, such as exercise, pollutants, or diet,^1,2^ and give detailed information related to metabolic pathways and biological processes. The advancement of analytical technologies in the field of metabolomics has made it possible to perform high-throughput metabolite identification and quantification in biological specimens. Furthermore, mass-spectrometry-based platforms have recently been used to characterize the human metabolome^3,4^ and to investigate the effect of diet on chronic diseases in many epidemiologic studies.^5,6^ Therefore, the application of metabolomics holds great promise for use in epidemiologic studies to identify potential biomarkers for estimating chronic disease risk. In the context of nutritional epidemiology, metabolomics now offers great promise, with many applications for both targeted and untargeted approaches. Untargeted metabolomics offers opportunities for the identification of novel biomarkers of food consumption and the identification of biomarkers of risk. Targeted metabolomic platforms also offer the possibility of identification of risk biomarkers and understanding the impact of diet on metabolic pathways.

Issues relevant to sample collection and storage, experimental assay errors, and within-person variance over time cause error and bias when measuring metabolites and identifying biomarkers.^7^ Furthermore, in many large epidemiologic studies, a single measurement is usually obtained because of limited resources and limited biological samples. To draw inference from a single measurement per individual, the within-subject variance of that metabolite over time should be known. Caution should be taken when interpreting metabolites with high variance. Additionally, poor reproducibility of metabolites may bias relative risk according to one measurement toward the null and undermine the potential use of metabolomics for identifying metabolic signatures related to diet or disease in human populations. Consequently, there is an urgent need to define the reproducibility of measurements at the metabolite level. Studies that estimate within-person variability over time are also necessary to determine whether a single measurement available in most epidemiologic studies could reflect the medium- or long-term levels. Previous studies indicate fair to excellent reproducibility for certain metabolites.^7−10^ For example, Carayol et al. (2015) reported a median intraclass correlation coefficient (ICC) value of 0.70 for 158 metabolites quantified in fasting serum samples collected 2 years apart from 27 healthy men, and 73% of metabolites showed ICC values >0.5. The study concluded that a single measurement per individual may be sufficient for those metabolites.^8^ In another similar study, two fasting serum samples were collected 4 months apart from healthy individuals (*n* = 100) from the European Prospective Investigation into Cancer and Nutrition (EPIC)-Potsdam study. There were 163 metabolites quantified, and the median ICC value was 0.57, which indicated that the reliability of serum metabolites over a 4 month period was good.^9^ However, these studies are limited by a small number of participants or limited repeated samples, making it necessary to examine the reproducibility across a larger population group with multiple sample repeats. Therefore, the objective of the present research was to evaluate the reproducibility of targeted plasma metabolites at four different time points over a 4 month period.

## Materials and Methods

### A-DIET Confirm Study

#### Study Outline

The A-DIET Confirm study was designed to examine the habitual dietary intake of participants during a period of 4 months. The detailed study information was previously described.^11^ In brief, ethical approval was granted by University College Dublin Sciences Human Research Ethics Committee (LS-16-91-Gibbons-Brennan). Healthy males and females, aged between 18 and 60 years old, with a body mass index (BMI) in the range of 18.5–30 kg/m^2^ and not consuming any supplements or prescribed medication were recruited. Participants who had any diagnosed health condition or were pregnant or lactating were excluded from this study. Following informed consent, participants were asked to attend an intervention suite at the Institute of Food and Health at University College Dublin once a month for four consecutive months, preferably in the same period of each month. Anthropometric data such as weight, height, and waist and hip circumference were measured in duplicate during each study visit. Dietary data and biological samples were collected at each visit.

#### Sample Collection

Fasting blood (6 mL) was collected via venipuncture by a trained phlebotomist using a lithium heparin tube. The sample tubes were inverted eight to ten times upon collection to mix the coagulant throughout and were placed on ice immediately. Subsequently, 500 μL aliquots of plasma were collected by centrifugation at 1800*g* for 10 min at 4 °C and were stored at −80 °C until further analysis. The blood samples from all visits were collected and processed according to this standardized procedure.

#### Dietary Data Collection

A 24 h dietary recall based on the U.S. Department of Agriculture Automated Multiple-Pass Method and also following the protocol recommended by Moshfegh et al. (2008),^12^ was used to collect dietary data at each visit. The portion sizes were verified by a photographic food atlas when the accurate amount of consumed food was not known. All of the 24 h dietary recalls were coded based on the food atlas.

Dietary intake data were entered into Nutritics (Dublin, Ireland), a software for dietary analysis, by two researchers independently and were also cross-checked for any discrepancies. A total of 31 food groups were defined based on previous studies, and each food or drink item was assigned to one of these food groups,^13,14^ which included: rice, pasta, and grains; savories; white bread rolls and scones; brown bread and wholemeal; breakfast cereals and porridge; biscuits, cakes, and pastries; whole milk; low-fat milk and skimmed milks; other milks, milk-based beverages, and other beverages; cream, ice creams, and desserts; cheese; yogurts; eggs and egg dishes; butter, fat spreads, and hard cooking fats; low-fat spreads and oils; potatoes; chips and processed potatoes; vegetables and vegetable dishes; fruit juices and smoothies; fruit; savory snacks; fish, fish dishes, and products; unprocessed red meat; unprocessed white meat; processed meats; alcoholic beverages; sugar syrups, preserves, and sweeteners; confectionary; soups, sauces, and condiments; low-energy beverages; and high-energy beverages. The data for each food group were reported as the percentage total energy (% TE), and prior to statistical analysis, the data were Z-score-transformed. For the present study, the average dietary data from the four 24 h dietary recalls and from 170 participants were included.

### Metabolomic Analysis

#### Sample Preparation

Plasma samples were collected and analyzed for targeted metabolomics. They were prepared and measured according to the AbsoluteIDQ p180 assay manual (Biocrates Life Sciences, Innsbruck, Austria). All plasma samples from the same individual were prepared identically and in the same batch with positions in the assay plate randomized. Ten μL of sample (plasma, pooled plasma, Biocrates quality controls (QCs), phosphate-buffered saline (PBS), or calibration standard solution) was added onto the filter inserts of the 96-well plate and then dried for 30 min at room temperature. Then, 50 μL of derivatization solution (5% phenyl isothiocyanate in ethanol/water/pyridine (volume ratio 1/1/1)) was added to the plate and incubated for 25 min at room temperature. The plate was subsequently dried for 60 min under a stream of nitrogen. Metabolites were extracted with 300 μL of 5 mM ammonium acetate in methanol by shaking the plate for 30 min and then centrifuged at 500*g* for 2 min. The eluate (150 μL) was diluted by adding 150 μL of high-performance liquid chromatography (HPLC)-grade water for running liquid chromatography tandem mass spectrometry (LC-MS/MS). For flow injection analysis tandem mass spectrometry (FIA-MS/MS) analysis, 50 μL of eluate was diluted with 450 μL of running solvent.

#### Sample Analysis by LC-MS

The AbsoluteIDQ p180 kit was prepared and then analyzed by a Sciex QTRAP 6500+ mass spectrometer coupled to Sciex ExionLC series UHPLC capability. During the LC-MS/MS run, metabolites were separated on a UHPLC column provided with an AbsoluteIDQ p180 kit using water with 0.2% formic acid and acetonitrile with 0.2% formic acid as mobile phase A and B, respectively. Amino acids (*n* = 21) and biogenic amines (*n* = 21) were quantified in the positive mode. For the FIA-MS/MS analyses, methanol was used as the running solvent, and 40 acylcarnitines, 14 lysophosphatidylcholines (lysoPC), 38 acyl/acyl phosphatidylcholines (PC aa), 38 acyl/alkyl phosphatidylcholines (PC ae), 15 sphingomyelins (SMs), and the sum of hexoses (H1) were identified and quantified in positive mode. All metabolites were quantified using multiple reaction monitoring (MRM), which was optimized and provided by Biocrates Life Sciences. Data acquisition was conducted by AB Sciex Analyst version 1.7.2 software.

#### Data Processing

The quantification of amino acids and biogenic amines was performed based on isotopically labeled internal standards and seven-point calibration curves in AB Sciex Analyst version 1.7.2 software. Other metabolites, such as acylcarnitines, lysoPCs, PCs, SMs, and hexose, were measured semiquantitatively by using 14 internal standards. The data quality was evaluated within the MetIDQ software, which was provided with the p180 kit, by checking the accuracy and reproducibility of QC samples. Normalized metabolite concentrations are reported in micromoles. Metabolites were included for further statistical analyses only when the concentrations of the metabolites were above the limit of detection (LOD) in >75% of plasma samples.

### Statistical Analyses

Data were tested for normality and subsequently log-transformed before analysis. Repeated-measures ANOVA was used to investigate the changes in the plasma metabolite across the visits using SPSS 24.0. Multiple comparisons were adjusted by the Benjamini–Hochberg procedure in R (version 4.0.2) using the p.adjust function. Adjusted *P* values of <0.05 were considered statistically significant. The reproducibility of metabolites was evaluated by ICCs calculated in SPSS 24.0 using a two-way mixed model with consistency and single measures reported,^15^ and the values were between 0 and 1. An ICC ≥ 0.75 was considered to represent excellent reproducibility, 0.60–0.75 to represent good reproducibility, 0.4–0.59 to represent fair reproducibility, and <0.40 to represent poor reproducibility.^16,17^ Hierarchical clustering analysis was applied to discover groupings among the metabolite data matrix from all four visits and to assess the similarity and dissimilarity between observations. Additionally, regularized canonical correlation analysis (rCCA) was performed to examine the potential factors influencing the metabolites across the four visits in R (version 4.0.2) using the mixOmics package. Furthermore, the correlation network was built with a threshold of 0.28.

## Results

### Plasma Metabolite Levels Are Highly Reproducible Across Multiple Time Points

Table 1 highlights the characteristics of participants with metabolomics data for the first visit of the A-DIET Confirm study: 55 men and 131 women were included with an average age of 35 years old and an average BMI of 24.00 ± 3.04 kg/m^2^. Blood samples were available for a total of 172 participants who completed two visits, 160 participants who completed three visits, and 141 participants who completed four visits. The analysis of the pooled plasma QC sample revealed that a high proportion (∼87%) of the metabolites had an interplate coefficient of variation (CV) < 20%, with 68 metabolites exhibiting CVs < 10% (Figure S1).

**Table 1:** Characteristics of Participants in the A-DIET Confirm Study^a^

characteristics	A-DIET Confirm study
gender	55(M)/131(F)
age (years)	35 ± 13
BMI at first visit (kg/m^2^)	24.00 ± 3.04

a Values are presented as the mean ± SD. Data are for participants with metabolomics data for visit 1.

A total of 138 plasma metabolites were measured including 20 amino acids, 11 biogenic amines, 10 acylcarnitines, 10 lysoPCs, 72 PCs, 14 SMs, and 1 hexose. The concentrations of each metabolite in each visit are shown in Table S1. Repeated-measures ANOVA was conducted to evaluate the significant difference in metabolites across four visits. From the amino acids and the biogenic amines, no metabolites showed a significant difference across four visits (false discovery rate (FDR)-adjusted *P* value >0.05) (Table 2). Furthermore, the ICC analysis revealed that all of the amino acids, except for methionine and valine, had ICC ≥ 0.4, and the median ICC was 0.49. For biogenic amines, the median ICC was 0.65, with the highest ICC of 0.75 for α-aminoadipic acid and the lowest ICC of 0.37 for serotonin.

**Table 2:** Analysis of Amino Acids and Biogenic Amines Across Four Visits (*n* = 141)^a^

metabolites	*P* value^b^	FDR-adjusted^c^	ICC	95% CI
Amino Acids				
alanine	0.3556	0.4931	0.43	0.35–0.52
arginine	0.0265	0.1164	0.40	0.31–0.49
asparagine	0.8299	0.8912	0.40	0.31–0.49
citrulline	0.2067	0.3356	0.62	0.54–0.69
glutamine	0.0401	0.1456	0.42	0.34–0.51
glutamate	0.0148	0.0966	0.57	0.49–0.65
glycine	0.1002	0.2248	0.62	0.55–0.69
histidine	0.1456	0.2692	0.44	0.36–0.53
isoleucine	0.3645	0.4931	0.53	0.45–0.62
leucine	0.3645	0.4931	0.52	0.44–0.60
lysine	0.1501	0.2726	0.54	0.46–0.62
methionine	0.2533	0.3797	0.33	0.24–0.42
ornithine	0.0754	0.2001	0.55	0.47–0.63
phenylalanine	0.2246	0.3444	0.44	0.35–0.53
proline	0.0737	0.2001	0.71	0.65–0.77
serine	0.0191	0.1012	0.58	0.50–0.65
threonine	0.3867	0.5131	0.44	0.36–0.53
tryptophan	0.4378	0.5500	0.45	0.36–0.54
tyrosine	0.0931	0.2178	0.55	0.47–0.53
valine	0.2887	0.4150	0.38	0.30–0.48
Biogenic Amines				
acetylornithine	0.8553	0.9024	0.66	0.59–0.73
asymmetric dimethylarginine	0.0892	0.2160	0.67	0.60–0.73
α-aminoadipic acid	0.7056	0.7728	0.75	0.69–0.80
creatinine	0.8566	0.9024	0.74	0.68–0.79
kynurenine	0.9391	0.9671	0.65	0.58–0.72
putrescine	0.0479	0.1653	0.50	0.41–0.58
sarcosine	0.1058	0.2248	0.71	0.65–0.77
symmetric dimethylarginine	0.2934	0.4174	0.63	0.55–0.70
serotonin	0.0177	0.1012	0.37	0.28–0.46
*trans*-4-hydroxyproline	0.1463	0.2692	0.46	0.38–0.55
taurine	0.6355	0.7116	0.55	0.47–0.63

a CI: confidence interval; ICC: intraclass correlation coefficient.

b *P* values are from repeated-measures ANOVA.

c FDR-adjusted *P* values are adjusted for multiple comparisons by the Benjamini–Hochberg procedure.

Ten short- or medium-chain acylcarinitines were measured with a median ICC of 0.69 (Table 3). The highest ICC value was 0.79 for C4, and the lowest ICC value was found for C2 (ICC = 0.51). The median ICC values for lysoPCs, PCs, and SMs were 0.66, 0.63, and 0.63, respectively. Nine out of ten lysoPCs, 53% of PCs, and 57% of SMs had ICC ≥ 0.6. Among these lipids that were poorly reproducible (ICC < 0.4), there was 1 out of 10 lysoPCs (lysoPC a C28:0, ICC = 0.32) and 17 out of 72 PCs and SM C18:0 (ICC = 0.22) and SM C18:1 (ICC = 0.30). The ICC for hexoses (H1) was 0.37. Overall, the median ICC of the 138 metabolites was 0.62, and 17% of metabolites were found to have ICC values <0.40, 29% of metabolites showed ICC values between 0.40 and 0.59, 40% of metabolites had ICC values between 0.60 and 0.75, and 14% of metabolites had ICC values ≥0.75. Furthermore, the majority of lipids did not significantly change across the four visits: Only one acylcarnitine (C2) and four lipids including one lysoPC (lysoPC a C18:2) and three PCs (PC aa C38:6, PC ae C32:1, PC ae C38:1) were significantly different across the four visits (Table 3). The impact of sex and age (age < 45 years; age ≥ 45 years) was also investigated. A total of 63 metabolites were significantly different between males and females, and 87 metabolites were significantly different between the two age groups (FDR-adjusted *P* value <0.05). (See Table S2.)

**Table 3:** Analysis of Acylcarnitines, Lipids, and Hexose across Four Visits (*n* = 141)^a^

metabolites	*P* value^b^	FDR-adjusted^c^	ICC	95% CI
Acylcarnitines				
C0	0.1049	0.2248	0.76	0.70–0.81
C2	0.0010	0.0331	0.51	0.43–0.59
C3	0.0127	0.0922	0.73	0.67–0.79
C4	0.8707	0.9034	0.79	0.74–0.83
C6 (C4:1-DC)	0.4683	0.5669	0.74	0.68–0.80
C8	0.6985	0.7711	0.71	0.65–0.77
C10	0.4372	0.5500	0.67	0.60–0.73
C10:1	0.6394	0.7116	0.60	0.52–0.67
C14:1	0.2559	0.3797	0.65	0.58–0.72
C18:1	0.4570	0.5631	0.66	0.59–0.73
lysoPhosphatidylcholines				
lysoPC a C16:0	0.1959	0.3257	0.68	0.61–0.74
lysoPC a C16:1	0.2191	0.3422	0.61	0.54–0.69
lysoPC a C17:0	0.1457	0.2692	0.74	0.69–0.80
lysoPC a C18:0	0.0867	0.2160	0.64	0.56–0.71
lysoPC a C18:1	0.0060	0.0753	0.65	0.58–0.72
lysoPC a C18:2	0.0006	0.0331	0.69	0.63–0.75
lysoPC a C20:3	0.0892	0.2160	0.65	0.58–0.72
lysoPC a C20:4	0.0206	0.1015	0.66	0.58–0.72
lysoPC a C28:0	0.6199	0.7116	0.32	0.23–0.41
lysoPC a C28:1	0.9975	0.9975	0.85	0.82–0.89
Phosphatidylcholines				
PC aa C24:0	0.6312	0.7116	0.31	0.23–0.41
PC aa C28:1	0.0526	0.1728	0.84	0.80–0.87
PC aa C30:0	0.4388	0.5500	0.50	0.41–0.58
PC aa C32:0	0.0728	0.2001	0.20	0.12–0.29
PC aa C32:1	0.2277	0.3453	0.46	0.37–0.54
PC aa C32:2	0.8331	0.8912	0.37	0.28–0.46
PC aa C32:3	0.0599	0.1857	0.57	0.50–0.65
PC aa C34:1	0.4424	0.5500	0.22	0.14–0.31
PC aa C34:2	0.4638	0.5664	0.15	0.07–0.24
PC aa C34:3	0.5461	0.6333	0.40	0.31–0.49
PC aa C34:4	0.9465	0.9675	0.58	0.50–0.66
PC aa C36:0	0.0544	0.1746	0.78	0.73–0.83
PC aa C36:1	0.2806	0.4119	0.59	0.51–0.66
PC aa C36:2	0.0876	0.2160	0.21	0.13–0.30
PC aa C36:3	0.4058	0.5318	0.22	0.14–0.31
PC aa C36:4	0.1075	0.2248	0.77	0.71–0.82
PC aa C36:5	0.0330	0.1231	0.69	0.62–0.75
PC aa C36:6	0.2143	0.3422	0.69	0.62–0.75
PC aa C38:0	0.0312	0.1208	0.81	0.77–0.85
PC aa C38:1	0.0984	0.2248	0.42	0.33–0.51
PC aa C38:3	0.3805	0.5098	0.22	0.14–0.31
PC aa C38:4	0.0270	0.1164	0.31	0.22–0.40
PC aa C38:5	0.0198	0.1012	0.71	0.64–0.77
PC aa C38:6	0.0002	0.0259	0.78	0.73–0.83
PC aa C40:2	0.1855	0.3160	0.45	0.36–0.54
PC aa C40:3	0.0054	0.0745	0.26	0.17–0.35
PC aa C40:4	0.6319	0.7116	0.68	0.61–0.74
PC aa C40:5	0.3595	0.4931	0.68	0.61–0.75
PC aa C40:6	0.0072	0.0764	0.77	0.72–0.82
PC aa C42:0	0.0141	0.0966	0.61	0.54–0.68
PC aa C42:1	0.4295	0.5500	0.48	0.39–0.56
PC aa C42:2	0.9711	0.9782	0.55	0.47–0.63
PC aa C42:4	0.1056	0.2248	0.38	0.29–0.47
PC aa C42:5	0.5384	0.6309	0.26	0.17–0.35
PC aa C42:6	0.1225	0.2415	0.59	0.51–0.66
PC ae C30:0	0.4085	0.5318	0.65	0.57–0.71
PC ae C30:1	0.8101	0.8803	0.23	0.15–0.32
PC ae C30:2	0.4786	0.5743	0.72	0.66–0.78
PC ae C32:1	0.0012	0.0331	0.74	0.68–0.79
PC ae C32:2	0.0096	0.0779	0.78	0.73–0.83
PC ae C34:0	0.2207	0.3422	0.75	0.70–0.81
PC ae C34:1	0.1397	0.2678	0.69	0.62–0.75
PC ae C34:2	0.0704	0.2001	0.68	0.61–0.74
PC ae C34:3	0.2159	0.3422	0.29	0.20–0.38
PC ae C36:0	0.1764	0.3045	0.68	0.61–0.74
PC ae C36:1	0.0924	0.2178	0.75	0.69–0.80
PC ae C36:2	0.2018	0.3315	0.74	0.68–0.79
PC ae C36:3	0.0734	0.2001	0.66	0.59–0.73
PC ae C36:4	0.1673	0.2960	0.70	0.63–0.76
PC ae C36:5	0.0309	0.1208	0.76	0.70–0.81
PC ae C38:0	0.0051	0.0745	0.71	0.65–0.77
PC ae C38:1	0.0008	0.0331	0.18	0.10–0.27
PC ae C38:2	0.1072	0.2248	0.62	0.55–0.69
PC ae C38:3	0.1194	0.2388	0.70	0.64–0.76
PC ae C38:4	0.0315	0.1208	0.73	0.67–0.79
PC ae C38:5	0.0087	0.0779	0.69	0.62–0.75
PC ae C38:6	0.0039	0.0745	0.79	0.74–0.84
PC ae C40:1	0.0612	0.1857	0.66	0.59–0.73
PC ae C40:2	0.0051	0.0745	0.69	0.62–0.75
PC ae C40:3	0.0521	0.1728	0.70	0.64–0.76
PC ae C40:4	0.0107	0.0820	0.50	0.42–0.59
PC ae C40:5	0.0093	0.0779	0.69	0.62–0.75
PC ae C40:6	0.0041	0.0745	0.79	0.74–0.84
PC ae C42:1	0.9623	0.9765	0.51	0.43–0.59
PC ae C42:2	0.5176	0.6158	0.45	0.36–0.54
PC ae C42:3	0.1280	0.2488	0.39	0.30–0.48
PC ae C42:4	0.1765	0.3045	0.63	0.56–0.70
PC ae C42:5	0.0853	0.2160	0.71	0.65–0.77
PC ae C44:3	0.0197	0.1012	0.34	0.26–0.44
PC ae C44:4	0.5395	0.6309	0.54	0.46–0.62
PC ae C44:5	0.1130	0.2327	0.44	0.36–0.53
PC ae C44:6	0.3409	0.4800	0.44	0.36–0.53
Sphingomyelins				
SM (OH) C14:1	0.0258	0.1164	0.89	0.85–0.91
SM (OH) C16:1	0.0184	0.1012	0.45	0.36–0.54
SM (OH) C22:1	0.0264	0.1164	0.73	0.67–0.79
SM (OH) C22:2	0.0072	0.0764	0.79	0.74–0.84
SM (OH) C24:1	0.1882	0.3167	0.45	0.36–0.54
SM C16:0	0.0094	0.0779	0.66	0.59–0.72
SM C16:1	0.0751	0.2001	0.75	0.70–0.80
SM C18:0	0.1606	0.2878	0.22	0.14–0.31
SM C18:1	0.0619	0.1857	0.30	0.21–0.39
SM C20:2	0.0288	0.1204	0.54	0.46–0.62
SM C24:0	0.1148	0.2330	0.64	0.57–0.71
SM C24:1	0.0154	0.0966	0.61	0.53–0.68
SM C26:0	0.8653	0.9034	0.44	0.36–0.53
SM C26:1	0.0449	0.1589	0.67	0.61–0.74
hexose (H1)	0.2868	0.4150	0.37	0.28–0.46

a Abbreviations are as follows. C*x*:*y*: *x* = number of carbons in the fatty acid side chain, *y* = number of double bonds in the fatty acid side chain; DC: decarboxyl; OH: hydroxyl; lysoPC: lysophosphatidylcholine; PC: phosphatidylcholine; aa: acyl–acyl; ae: acyl–alkyl; SM: sphingomyelin; CI: confidence interval; ICC: intraclass correlation coefficient.

b *P* values are from repeated-measures ANOVA.

c FDR-adjusted *P* values are adjusted for multiple comparisons by the Benjamini–Hochberg procedure.

### Samples from Individuals Cluster Together

Hierarchical clustering trees were constructed to examine the grouping of each participant at the visits (Table 4). In total, 160 participants had metabolite data for a minimum of three visits (Table 4). When these data were considered, a total of 113 subjects grouped together for their data. When participants with data for ≥2 visits (*n* = 172) were examined, 90.12% clustered with their data from the other visits.

**Table 4:** Identification of Participants Who Were Distributed into the Same Group by Hierarchical Clustering Analysis

no. of participants ≥3 visits	no. of participants ≥3 visits grouping together	percentage
160	113	70.63%

no. of participants ≥2 visits	no. of participants ≥2 visits grouping together	percentage
172	155	90.12%

**Figure 1:**
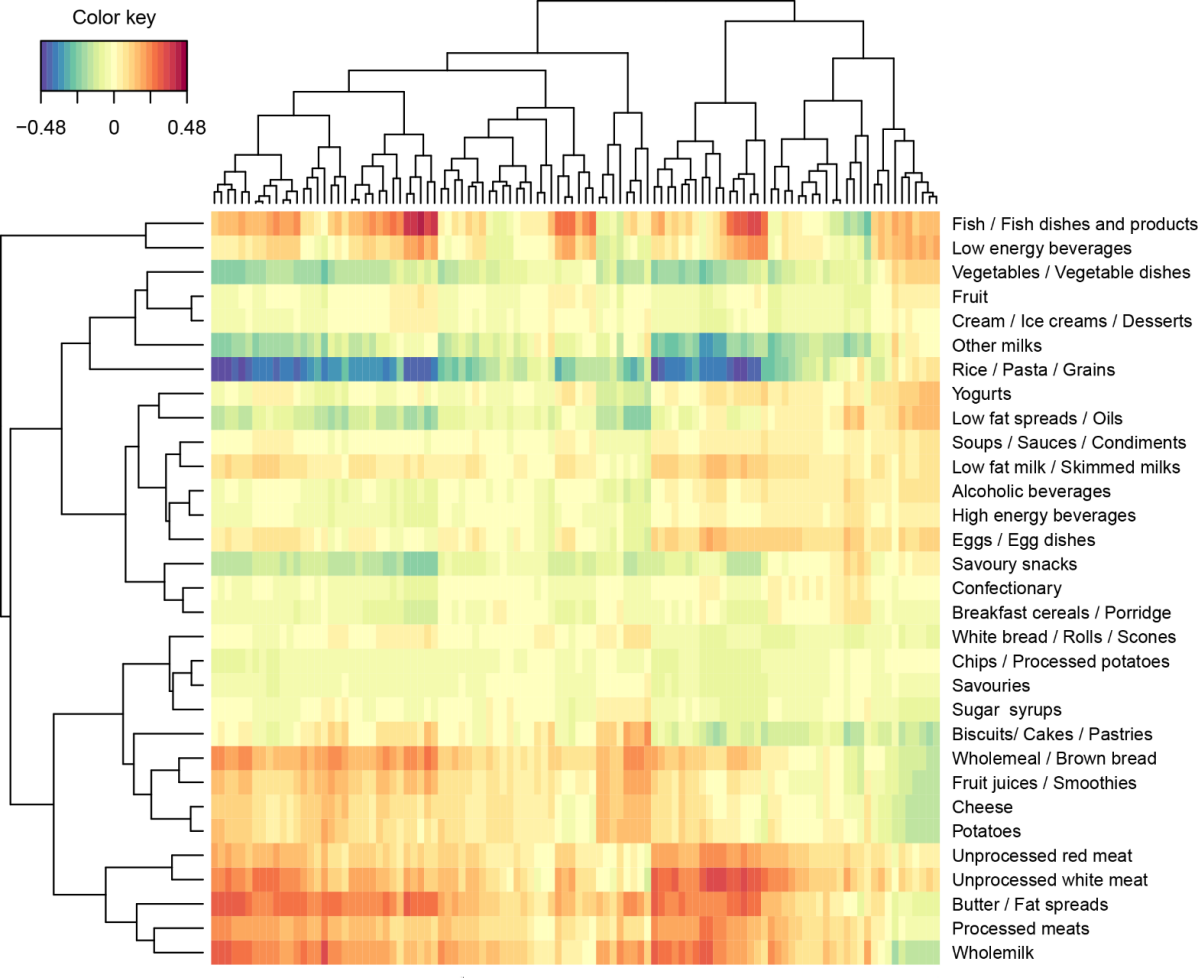
Heat map of output from regularized canonical correlation analysis (rCCA) examining the association between lipid profiles and food intake (% total energy). The *x* axis represents the measured lipids, and the *y* axis represents the food intake. Correlation strengths are indicated by the color key. Other milks: other milks, milk-based beverages, and other beverages; Butter/Fat spreads: butter, fat spreads, and hard cooking fats; Sugar: sugar syrups, preserves, and sweeteners.

**Figure 2:**
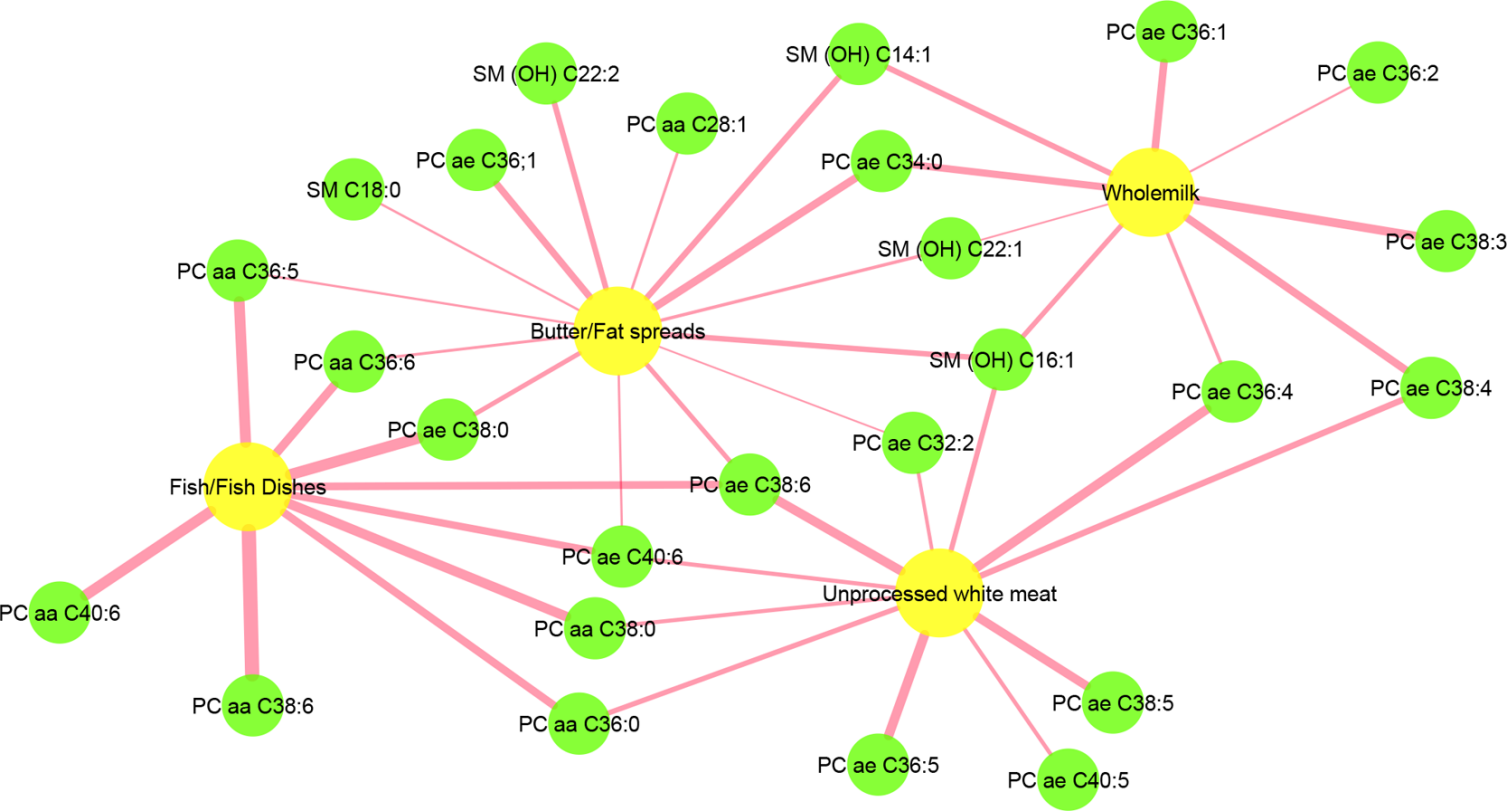
Network graph depicting the positive correlations derived from regularized canonical correlation analysis (rCCA) between lipid profiles and food intake (% total energy) with correlation coefficient >0.28. The edge is sized according to the correlation strengths, with a wider edge indicating a higher correlation. Butter/Fat spreads: butter, fat spreads, and hard cooking fats; Fish/Fish dishes: fish, fish dishes, and products. PC, phosphatidylcholine. SM, sphingomyelin.

To further examine the potential factors influencing the metabolites across the visits, we performed a regularized canonical correlation analysis (rCCA) to examine the correlations between lipid profiles and food intake data (lipid data from visit 1 and food intake data from the average of four 24 h dietary recalls). Whole milk, butter/fat spread, unprocessed white meat, and fish and fish dishes showed positive correlations with some lipids (Figure 1). To clarify the correlation coefficients in detail, we built a network that displays the interaction between lipids and food intake (Figure 2). Whole milk intake was positively correlated with nine lipids, including six PCs and three SMs; butter/fat spread intake was positively correlated with nine PCs and five SMs; fish and fish dish intake showed a positive correlation with nine PCs; and unprocessed white meat intake was positively correlated with ten PCs and one SM.

## Discussion

Our results indicate that metabolite levels measured using a targeted LC-MS/MS metabolomics platform resulted in highly reproducible data in a healthy adult population over a 4 month period. Reproducibility was fair to good for the majority of amino acids and biogenic amines. The majority of short- and medium-chain acylcarnitines and most phosphatidylcholines and sphingomyelins showed good to excellent reproducibility. Consequently, for these metabolites within-subject variation is low, and a single measurement could reflect their concentrations appropriately and may also be sufficient for risk assessment in epidemiologic studies in a free-living population.

Examination of ICC values is a useful tool for assessing reproducibility, as both between- and within-person variability are considered. Several previous studies have evaluated the metabolite reproducibility over a time period through the ICC.^8−10^ For example, Floegel et al. (2011) evaluated the serum metabolite reproducibility (*n* = 163) in 100 healthy participants (50 men and 50 women) from the cohort of the European Prospective Investigation into Cancer and Nutrition (EPIC)-Potsdam study over a period of 4 months. Two fasting blood samples 4 months apart were collected and analyzed using the Biocrates AbsoluteIDQ p150 kit. Fair to excellent reproducibility for most metabolites was reported, with a median ICC of 0.57 for the 163 metabolites measured.^9^ Another study included 39 healthy women with nonfasting blood samples on two occasions at a 2.4 year interval and 27 healthy men with fasting blood samples on two occasions at a 1.9 year interval. The results reported a median ICC value of 0.70 among 158 metabolites measured in fasting serum samples, and 73 and 52% of metabolites showed ICC > 0.50 in fasting and nonfasting serum samples, respectively.^8^ Our study involved a higher number of participants and multiple sample collections over a 4 month period and demonstrated similar ICC values for those metabolites when compared with the studies mentioned above.

The good reproducibility of measured metabolites was further explored by hierarchical clustering analysis. Interestingly, the majority of participants were clustered together based on their metabolomic data. Taken together with the ICC data, one could make the case for the development of defined reference ranges of metabolites for healthy individuals. These reference ranges could be used to track a person’s metabolites over time and importantly to identify metabolite deviations from normal, which may be indicative of early perturbations to metabolism prior to disease development.

In the literature, amino acid levels measured in blood samples generally display good reproducibility.^9,10^ One reason could be that amino acids in plasma are not particularly influenced by a different nutritional status, and genetic regulation plays an important role in their homeostasis.^18^ Therefore, the concentration levels of amino acids for intraindividuals are within a narrow range. In plasma and serum samples, acylcarnitines are generally observed at low concentrations. Several saturated or monounsaturated short- and medium-chain acylcarinitines were quantified and exhibited good to excellent reproducibility in the fasting plasma in the present study. Breier et al. (2014) also reported that the reproducibility of acylcarnitine was good for most saturated short- and medium-chain acylcarnitines, as measured in fasting serum and plasma samples from 22 healthy participants.^10^ However, it is worth noting that in our study, most acylcarnitines revealed higher interplate variability in pooled QC samples, which implies the need for extra attention when using them in epidemiological studies.

PCs and SMs belong to the group of membrane phospholipids, and lysoPCs are mainly derived from the partial hydrolysis of PCs via the lipoprotein-associated phospholipase A2.^19^ The synthesis and redistribution between PCs and lysoPCs could impact their concentrations in blood. The present study indicated that the majority of lysoPCs, PCs, and SMs measured were highly reproducible over 4 months. Findings from previous studies corroborate these findings. In two samples from 100 individuals in a 4 month period, the reproducibility was high for serum sphingolipids (median ICC = 0.66; range: 0.24–0.85) and glycerophospholipids (median ICC = 0.58; range: 0.03–0.81).^9^ In another study, 158 metabolites in fasting serum samples were evaluated for reproducibility over a 2 year period, and lysoPCs (median ICC = 0.63; range: 0.39–0.79), PCs (median ICC = 0.74; range: 0.43–0.91), and SMs (median ICC = 0.77; range: 0.54–0.88) all indicated good to excellent reproducibility.^8^ Additionally, the reproducibility of platelet membrane phospholipids measured in 12 human participants over a 3 week period reported an ICC of 0.50 for total PCs and 0.58 for total SMs.^20^ Taken collectively, the evidence indicates a high reproducibility for PCs, lysoPCs, and SMs when measured in multiple samples from the same individuals.

To examine the potential factors influencing the metabolite, we further investigated correlations between lipids and food intake through rCCA. Positive correlations were found between whole milk, butter/fat spreads, unprocessed red meat, fish/fish dishes, and certain lipids. Previous studies indicated that dairy, red meat, and fish intake were directly correlated with blood lipids.^21−23^ It is interesting to note that despite the relationship with food intake, these metabolites had good reproducibility. The importance of this lies in the concept that blood metabolites can be influenced by exogenous factors such as diet, but the reproducibility within a person remains high. This supports the emerging concept of modeling individuals over time and the development of trajectories for monitoring and identifying early perturbations that may be indicators of disease risk.

Although the present study provides strong evidence of the reproducibility of metabolites, there are a few limitations worth noting. One of the limitations was the fact that the study was conducted over a 4 month interval; evaluating the stability over longer timeframes with multiple samples will be important for the future. The present study was performed in a healthy population, and metabolite reproducibility may be different in people with some chronic diseases or with a different nutritional status. Therefore, future studies are warranted to examine the metabolite reproducibility in different populations. There are also several strengths of our study. The main one was that we evaluated the reproducibility in a wide spectrum of metabolites including many different compound classes using a well-validated, high-throughput technique, which makes it possible to extend to large epidemiological studies. We report the concentrations for all metabolites, making it an important reference for future studies. Additionally, a healthy, free-living population who was exposed to many external stimuli was involved in this study; their metabolite information reflects real-life situations.

## Conclusions

The current study showed good to excellent reproducibility for most of the metabolites over four time points for a 4 month period. For those metabolites, a single assessment in epidemiologic studies could appropriately reflect their concentrations in individuals and may be sufficient for assessing the risk.

## Supporting Information Available

Figure S1. Distribution of % CV of metabolites quantified in pooled plasma QC sample. Table S1. Concentrations of metabolites in each visit. Table S2. Impact of sex and age groups on the metabolite levels (PDF)

## Notes

The study data are deposited in MetaboLights (MTBLS3428 (www.ebi.ac.uk/metabolights)).
