# Supplementary material for: Targeted Metabolomic Approach to Assess the Reproducibility of Plasma Metabolites over a Four Month Period in a Free-Living Population
Source: J Proteome Res. 2022 Jan 3;21(3):683–90. doi: 10.1021/acs.jproteome.1c00440 (PMC8902803; doi:10.1021/acs.jproteome.1c00440)
Supplement: Supplementary file 1 — pr1c00440_si_001.pdf [file pr1c00440_si_001.pdf]

SUPPORTING INFORMATION

A targeted metabolomic approach to assess the reproducibility of plasma metabolites over a four-month period in a free-living population

Xiaofei Yin[1,2], Orla Prendiville[1,2], Aoife E. McNamara[1,2], Lorraine Brennan*[,1,2]

[1] UCD School of Agriculture and Food Science, Institute of Food and Health, University College Dublin, Belfield, Dublin 4, D4 V1W8, Ireland

[2] UCD Conway Institute of Biomolecular and Biomedical Research, University College Dublin, Belfield, Dublin 4, D4 V1W8, Ireland

*Corresponding Author:

Professor Lorraine Brennan

School of Agriculture and Food Science,

Institute of Food and Health,

University College Dublin, Belfield, Dublin 4, Ireland.

Email: lorraine.brennan@ucd.ie

Phone: 00 353 1 7166815

TABLE OF CONTENTS

S2 **Figure S1.** Distribution of % CV of metabolites quantified in pooled plasma QC sample. lysoPC: lysophosphatidylcholine; PC: phosphatidylcholine; aa: acyl-acyl; ae: acyl-alkyl.

S3 **Table S1** Concentrations (μM) of metabolites in each visit.

S7 **Table S2** Impact of sex and age groups on the metabolite levels.

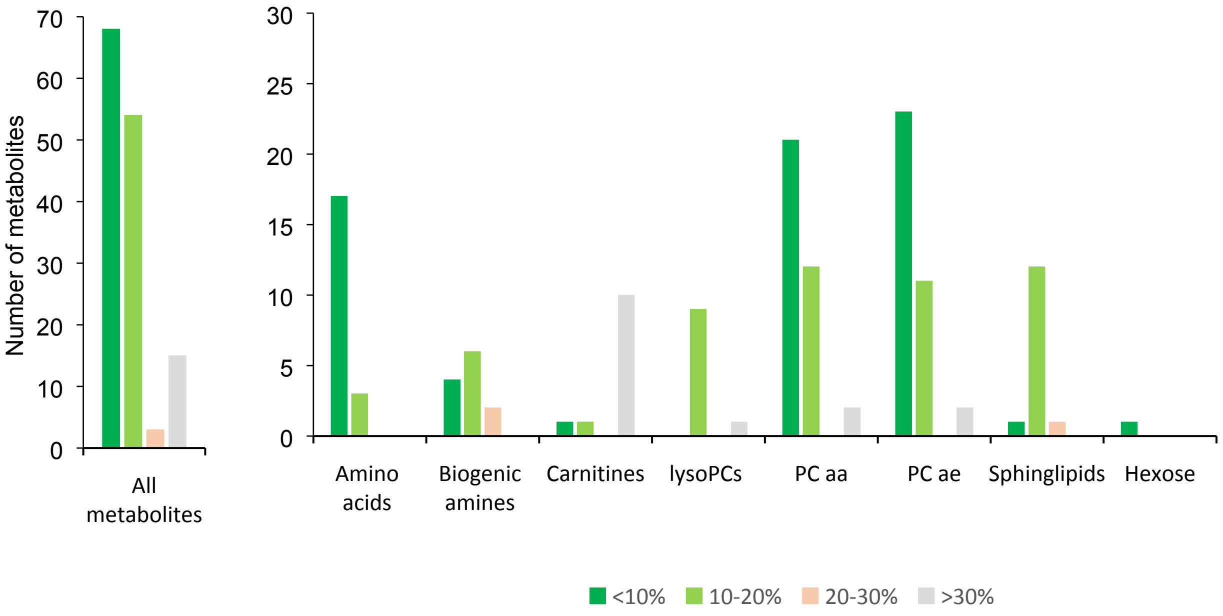

Figure S1. Distribution of % CV of metabolites quantified in pooled plasma QC sample. lysoPC: lysophosphatidylcholine; PC: phosphatidylcholine; aa: acyl-acyl; ae: acyl-alkyl.

Table S1 Concentrations (μM) of metabolites in each visit.

Metabolites	V1 (n=186)	V2 (n=166)	V3 (n=156)	V4 (n=151)
**Amino acids**				
Alanine	360.87±85.55	353.44±78.14	368.08±85.62	362.56±92.35
Arginine	101.43±20.18	99.4±19.41	103.27±20.03	100.39±19.64
Asparagine	48.43±8.66	49.96±28.89	48.13±10.32	47.46±8.02
Citrulline	31.93±7.34	31.95±7.21	32.33±7.01	32.3±7.28
Glutamine	716.37±90.98	722.25±91.01	720.34±83.2	705.59±92.48
Glutamate	30.86±18.72	31.9±18.27	35.17±19.00	35.33±18.65
Glycine	276.24±82.98	281.14±81.40	284.65±80.86	276.76±75.65
Histidine	105.13±12.17	105.11±12.96	105.53±12.24	103.32±12.02
Isoleucine	81.80±19.06	83.19±18.50	82.65±17.80	82.17±20.41
Leucine	174.28±37.64	176.63±36.63	174.93±34.75	172.97±39.05
Lysine	292.28±31.32	294.49±33.07	295.21±33.19	290.16±31.66
Methionine	28.74±5.58	28.84±5.30	29.09±5.32	28.64±5.36
Ornithine	75.69±15.42	75.36±16.21	77.01±15.68	75.41±16.30
Phenylalanine	102.46±11.89	102.93±11.86	103.11±10.98	101.61±12.06
Proline	180.99±56.94	185.92±56.77	184.03±53.30	184.28±67.34
Serine	126.04±27.63	127.68±26.07	128.52±24.26	125.96±25.56
Threonine	130.57±32.60	126.42±31.48	128.16±29.49	129.62±32.25
Tryptophan	85.83±12.83	85.76±13.10	86.04±12.56	84.76±12.75
Tyrosine	73.47±15.30	73.59±14.95	74.88±15.30	73.34±15.10
Valine	277.80±44.04	281.95±43.51	281.98±38.95	280.30±41.10
**Biogenic amines**				
Acetylornithine	1.15±1.11	1.3±1.27	1.22±0.92	1.24±1.37
Asymmetric dimethylarginine	0.48±0.08	0.48±0.08	0.49±0.08	0.48±0.08
alpha-Aminoadipic acid	0.63±0.28	0.64±0.26	0.63±0.27	0.63±0.24
Creatinine	75.11±16.48	76.14±17.03	75.61±16.94	75.69±16.87
Kynurenine	2.38±0.52	2.39±0.53	2.39±0.52	2.39±0.54
Putrescine	0.13±0.03	0.13±0.04	0.12±0.03	0.13±0.03
Sarcosine	1.31±0.51	1.33±0.47	1.36±0.46	1.38±0.52
Symmetric dimethylarginine	0.47±0.08	0.47±0.08	0.47±0.08	0.46±0.08
Serotonin	0.17±0.13	0.14±0.09	0.14±0.11	0.13±0.08
trans-4-Hydroxyproline	9.26±4.95	9.57±6.19	9.7±4.74	9.34±4.75
Taurine	53.33±12.43	52.99±11.34	54.06±13.65	52.85±12.39
**Acylcarnitines**				
C0	30.18±7.42	30.82±7.45	31.04±7.55	30.69±7.17
C2	6.64±2.28	6.78±2.57	6.13±2.05	6.14±2.00
C3	0.29±0.09	0.3±0.09	0.3±0.10	0.3±0.10
C4	0.23±0.29	0.22±0.13	0.23±0.20	0.22±0.15
C6 (C4:1-DC)	0.10±0.08	0.10±0.10	0.10±0.10	0.10±0.10
C8	0.30±0.28	0.31±0.30	0.32±0.30	0.32±0.30
C10	0.41±0.34	0.42±0.35	0.42±0.34	0.41±0.34

C10:1	0.28±0.18	0.29±0.20	0.29±0.19	0.29±0.20
C14:1	0.17±0.17	0.19±0.20	0.19±0.20	0.19±0.21
C18:1	0.13±0.08	0.14±0.09	0.14±0.10	0.14±0.10
**lysoPhosphatidylcholines**				
lysoPC a C16:0	59.38±14.81	60.84±14.65	60.96±14.41	59.64±15
lysoPC a C16:1	17.58±124.97	1.85±0.62	1.83±0.63	1.79±0.62
lysoPC a C17:0	1.23±0.43	1.26±0.44	1.29±0.46	1.23±0.44
lysoPC a C18:0	18.00±5.61	18.17±5.46	18.67±5.62	18.09±5.82
lysoPC a C18:1	15.18±5.31	15.98±5.37	15.95±5.39	15.66±5.18
lysoPC a C18:2	25.76±11.11	26.95±10.52	27.42±10.18	27.27±10.17
lysoPC a C20:3	1.56±0.59	1.64±0.62	1.65±0.67	1.64±0.64
lysoPC a C20:4	4.49±1.68	4.69±1.74	4.64±1.74	4.56±1.74
lysoPC a C28:0	0.14±0.03	0.14±0.03	0.14±0.03	0.14±0.03
lysoPC a C28:1	0.33±0.15	0.33±0.15	0.33±0.15	0.33±0.15
**Phosphatidylcholines**				
PC aa C24:0	0.07±0.03	0.07±0.05	0.07±0.03	0.07±0.03
PC aa C28:1	2.59±0.77	2.56±0.75	2.53±0.71	2.48±0.66
PC aa C30:0	3.42±1.38	3.39±1.37	3.29±1.19	3.22±1.1
PC aa C32:0	11.14±2.46	11±2.22	10.76±2.35	10.47±2.09
PC aa C32:1	11.01±5.76	11.28±6.05	10.69±5.51	10.55±5.51
PC aa C32:2	3.57±1.80	3.52±1.68	3.55±1.74	3.44±1.45
PC aa C32:3	0.36±0.10	0.35±0.10	0.37±0.11	0.35±0.09
PC aa C34:1	172.45±42.23	176.58±43.99	173.15±39.91	170.31±41.23
PC aa C34:2	300.58±57.8	302.17±55.45	302.78±52.06	298.91±52.11
PC aa C34:3	13.58±4.49	13.87±4.88	14.10±4.72	13.76±4.48
PC aa C34:4	1.25±0.58	1.24±0.55	1.24±0.54	1.21±0.46
PC aa C36:0	2.45±1.15	2.35±1.04	2.39±1.02	2.24±0.99
PC aa C36:1	42.07±11.75	43.06±11.95	43.44±11.2	42.37±11.27
PC aa C36:2	204.8±47.86	206.17±44.59	210.31±41.25	205.98±41.9
PC aa C36:3	111.07±26.5	112.74±30.49	112.87±26.97	111.11±25.14
PC aa C36:4	147.11±38.09	146.6±37	142.81±32.11	141.17±32.59
PC aa C36:5	21.79±12.73	21.85±11.41	21.77±10.85	19.76±8.89
PC aa C36:6	0.90±0.45	0.90±0.43	0.91±0.43	0.84±0.35
PC aa C38:0	2.65±0.84	2.66±0.86	2.68±0.85	2.54±0.81
PC aa C38:1	0.88±0.54	0.89±0.61	0.96±0.60	0.86±0.58
PC aa C38:3	36.12±10.86	36.16±10.81	36.43±10.57	35.4±10.24
PC aa C38:4	81.47±21.91	80.55±22.45	80.11±19.31	77.93±19.47
PC aa C38:5	43.31±12.32	43.38±11.44	42.89±10.68	41.14±10.65
PC aa C38:6	65.31±23.25	65.18±22.69	63.98±21.2	60.05±18.68
PC aa C40:2	0.22±0.06	0.22±0.06	0.23±0.07	0.22±0.06
PC aa C40:3	0.42±0.10	0.42±0.10	0.43±0.10	0.41±0.09
PC aa C40:4	2.26±0.59	2.25±0.59	2.22±0.57	2.19±0.59
PC aa C40:5	7.3±2.34	7.2±2.16	7.23±2.20	7.05±2.25
PC aa C40:6	21.04±7.91	20.83±7.66	20.8±7.19	19.75±6.86

PC aa C42:0	0.48±0.11	0.48±0.12	0.48±0.11	0.45±0.10
PC aa C42:1	0.17±0.04	0.17±0.04	0.17±0.04	0.17±0.04
PC aa C42:2	0.18±0.04	0.18±0.05	0.18±0.04	0.18±0.05
PC aa C42:4	0.12±0.03	0.12±0.03	0.12±0.02	0.12±0.03
PC aa C42:5	0.26±0.08	0.25±0.07	0.26±0.07	0.24±0.06
PC aa C42:6	0.41±0.13	0.40±0.12	0.4±0.10	0.38±0.10
PC ae C30:0	0.31±0.09	0.31±0.10	0.31±0.09	0.30±0.09
PC ae C30:1	0.09±0.06	0.09±0.06	0.1±0.08	0.09±0.06
PC ae C30:2	0.09±0.02	0.09±0.02	0.09±0.02	0.08±0.02
PC ae C32:1	2.41±0.54	2.38±0.53	2.42±0.53	2.31±0.52
PC ae C32:2	0.62±0.15	0.62±0.16	0.63±0.16	0.60±0.15
PC ae C34:0	1.16±0.39	1.17±0.37	1.17±0.38	1.12±0.35
PC ae C34:1	8.09±1.77	8.13±1.89	8.12±1.74	7.81±1.70
PC ae C34:2	10.36±2.52	10.27±2.66	10.56±2.58	10.18±2.48
PC ae C34:3	7.72±2.30	7.45±2.12	7.64±2.20	7.38±2.14
PC ae C36:0	0.68±0.16	0.68±0.16	0.68±0.16	0.66±0.15
PC ae C36:1	5.8±1.54	5.87±1.57	5.87±1.51	5.62±1.45
PC ae C36:2	12.57±3.22	12.55±3.23	12.86±3.21	12.3±2.92
PC ae C36:3	6.84±1.56	6.82±1.65	7.01±1.62	6.78±1.56
PC ae C36:4	15.47±4.61	15.24±4.12	15.44±4.12	14.98±4.28
PC ae C36:5	10.08±4.02	9.74±2.87	9.93±3.06	9.53±3.15
PC ae C38:0	2.36±0.84	2.37±0.87	2.42±0.84	2.25±0.73
PC ae C38:1	0.33±0.15	0.33±0.15	0.370.14	0.33±0.15
PC ae C38:2	1.29±0.31	1.30±0.33	1.34±0.33	1.28±0.31
PC ae C38:3	2.64±0.69	2.66±0.69	2.69±0.70	2.57±0.63
PC ae C38:4	10.96±2.65	10.87±2.47	10.92±2.47	10.45±2.27
PC ae C38:5	14.04±3.48	13.88±2.93	14.03±2.96	13.49±3.03
PC ae C38:6	6.42±2.09	6.36±1.94	6.45±1.93	6.10±1.94
PC ae C40:1	1.12±0.29	1.11±0.28	1.14±0.28	1.09±0.26
PC ae C40:2	1.37±0.41	1.37±0.40	1.37±0.38	1.30±0.36
PC ae C40:3	0.64±0.12	0.65±0.13	0.65±0.13	0.63±0.11
PC ae C40:4	1.53±0.29	1.51±0.30	1.53±0.29	1.47±0.27
PC ae C40:5	2.36±0.50	2.35±0.45	2.35±0.46	2.25±0.43
PC ae C40:6	3.95±1.19	3.96±1.17	3.97±1.15	3.73±1.05
PC ae C42:1	0.28±0.05	0.28±0.05	0.28±0.05	0.28±0.05
PC ae C42:2	0.48±0.11	0.48±0.11	0.49±0.12	0.47±0.11
PC ae C42:3	0.65±0.13	0.65±0.15	0.65±0.15	0.63±0.13
PC ae C42:4	0.66±0.14	0.66±0.15	0.66±0.14	0.64±0.14
PC ae C42:5	1.55±0.31	1.54±0.32	1.54±0.29	1.49±0.28
PC ae C44:3	0.07±0.01	0.07±0.01	0.07±0.01	0.07±0.01
PC ae C44:4	0.31±0.07	0.31±0.07	0.31±0.06	0.30±0.07
PC ae C44:5	1.47±0.38	1.47±0.38	1.47±0.33	1.42±0.33
PC ae C44:6	1.05±0.26	1.05±0.25	1.05±0.24	1.00±0.23
**Sphingomyelins**				

SM (OH) C14:1	6.57±2.08	6.55±2.00	6.49±1.85	6.29±1.77
SM (OH) C16:1	3.50±1.09	3.50±1.05	3.46±1.01	3.34±0.96
SM (OH) C22:1	12.67±3.39	12.62±3.23	12.46±2.93	12.07±2.73
SM (OH) C22:2	12.35±3.56	12.28±3.39	12.24±2.99	11.74±2.92
SM (OH) C24:1	1.24±0.35	1.24±0.32	1.23±0.31	1.19±0.30
SM C16:0	113.72±21.93	113.19±20.74	112.77±19.57	108.75±18.55
SM C16:1	15.77±3.37	15.66±3.29	15.59±3.08	15.18±3.03
SM C18:0	23.94±6.12	23.85±5.78	23.47±5.68	22.6±5.73
SM C18:1	11.41±3.09	11.45±3.05	11.29±2.8	10.94±2.74
SM C20:2	0.33±0.10	0.33±0.10	0.33±0.09	0.31±0.09
SM C24:0	20.32±4.44	20.24±4.54	19.87±3.96	19.46±3.91
SM C24:1	56.91±11.83	57.12±11.89	56.57±10.95	54.47±9.99
SM C26:0	0.11±0.03	0.11±0.04	0.11±0.03	0.11±0.03
SM C26:1	0.28±0.09	0.28±0.09	0.27±0.09	0.26±0.07
Hexose (H1)	4860.75±501.68	4907.77±565.99	4915.99±532.07	4855.54±494.15

Abbreviations are as follows. Cx:y: x=number of carbons in the fatty acid side chain, y=number of double bonds in the fatty acid side chain; DC: decarboxyl; OH: hydroxyl; lysoPC: lysophosphatidylcholine; PC: phosphatidylcholine; aa: acyl-acyl; ae: acyl-alkyl; SM: sphingomyelin.

Note: Amino acids and biogenic amines were quantified based on internal standards and calibration curves consisting of seven calibration standards, while acylcarnitines, LPCs, PCs, SMs and hexose were evaluated semi-quantitatively by using 13 internal standards for lipids and acylcarnitines and 1 for the hexose.

Table S2 Impact of sex and age groups on the metabolite levels.

Metabolites	P value (Sex) *	FDR adjusted (Sex) **	P value (Age) *	FDR adjusted (Age) **
**Amino acids**				
Alanine	0.7009	0.7677	0.9078	0.9211
Arginine	0.2033	0.2862	0.2031	0.2644
Asparagine	0.7205	0.7768	0.0007	**0.0017**
Citrulline	0.0322	0.0645	0.0387	0.0588
Glutamine	0.0129	**0.0307**	0.0014	**0.0031**
Glutamate	0.0004	**0.0026**	0.0000	**7.0769E-06**
Glycine	0.3734	0.4727	0.1145	0.1612
Histidine	0.5951	0.6844	0.8708	0.9070
Isoleucine	0.0000	**1.4556E-09**	0.5119	0.6143
Leucine	0.0000	**8.2241E-12**	0.8301	0.8853
Lysine	0.0859	0.1412	0.0015	**0.0034**
Methionine	0.0128	**0.0307**	0.3975	0.4898
Ornithine	0.0020	**0.0093**	0.0034	**0.0066**
Phenylalanine	0.0000	**0.0004**	0.5667	0.6410
Proline	0.0003	**0.0021**	0.6331	0.7046
Serine	0.1156	0.1772	0.1912	0.2513
Threonine	0.0440	0.0829	0.0099	**0.0173**
Tryptophan	0.0000	**0.0001**	0.2871	0.3668
Tyrosine	0.0038	**0.0138**	0.0554	0.0831
Valine	0.0000	**0.0001**	0.5448	0.6345
**Biogenic amines**				
Acetylornithine	0.6156	0.6923	0.9015	0.9211
Asymmetric dimethylarginine	0.0636	0.1070	0.0000	**1.0535E-06**
alpha-Aminoadipic acid	0.0000	**3.9750E-07**	0.0299	**0.0479**
Creatinine	0.0000	**6.4671E-17**	0.0868	0.1235
Kynurenine	0.0000	**7.5845E-09**	0.0100	**0.0173**
Putrescine	0.0127	**0.0307**	0.1787	0.2371
Sarcosine	0.0000	**0.0001**	0.8742	0.9070
Symmetric dimethylarginine	0.0000	**0.0002**	0.2053	0.2648
Serotonin	0.2611	0.3498	0.8209	0.8850
trans-4-Hydroxyproline	0.0481	0.0884	0.0706	0.1015
Taurine	0.9187	0.9391	0.0046	**0.0086**
**Acylcarnitines**				
C0	0.0000	**2.1551E-06**	0.0000	**0.0001**
C2	0.0532	0.0941	0.0338	0.0518
C3	0.0000	**9.9427E-06**	0.0005	**0.0012**
C4	0.0008	**0.0040**	0.6275	0.7040
C6 (C4:1-DC)	0.2194	0.3028	0.8039	0.8735
C8	0.1425	0.2161	0.5378	0.6343
C10	0.1845	0.2652	0.5208	0.6195
C10:1	0.0284	0.0594	0.6792	0.7442
C14:1	0.0117	**0.0299**	0.5563	0.6345

C18:1	0.0089	**0.0236**	0.1718	0.2302
**lysoPhosphatidylcholines**				
lysoPC a C16:0	0.0071	**0.0201**	0.0019	**0.0040**
lysoPC a C16:1	0.0071	**0.0201**	0.0001	**0.0004**
lysoPC a C17:0	0.0046	**0.0151**	0.0000	**9.3259E-08**
lysoPC a C18:0	0.0089	**0.0236**	0.0001	**0.0004**
lysoPC a C18:1	0.0100	**0.0262**	0.1497	0.2066
lysoPC a C18:2	0.0023	**0.0101**	0.5511	0.6345
lysoPC a C20:3	0.0000	**1.7250E-05**	0.0070	**0.0128**
lysoPC a C20:4	0.0000	**0.0004**	0.3857	0.4795
lysoPC a C28:0	0.3515	0.4534	0.0560	0.0831
lysoPC a C28:1	0.3611	0.4613	0.1569	0.2144
**Phosphatidylcholines**				
PC aa C24:0	0.8672	0.9066	0.0021	**0.0044**
PC aa C28:1	0.1873	0.2664	0.0000	**6.3963E-10**
PC aa C30:0	0.0004	**0.0027**	0.0006	**0.0015**
PC aa C32:0	0.0020	**0.0093**	0.0003	**0.0008**
PC aa C32:1	0.0127	**0.0307**	0.0013	**0.0029**
PC aa C32:2	0.0028	**0.0114**	0.0330	0.0512
PC aa C32:3	0.0058	**0.0178**	0.0000	**3.6662E-06**
PC aa C34:1	0.6171	0.6923	0.9046	0.9211
PC aa C34:2	0.7183	0.7768	0.4551	0.5525
PC aa C34:3	0.1534	0.2253	0.3159	0.3964
PC aa C34:4	0.0071	**0.0201**	0.0017	**0.0036**
PC aa C36:0	0.0876	0.1423	0.0012	**0.0027**
PC aa C36:1	0.9714	0.9785	0.0069	**0.0128**
PC aa C36:2	0.3005	0.3949	0.8340	0.8853
PC aa C36:3	0.0625	0.1065	0.0076	**0.0139**
PC aa C36:4	0.0295	0.0599	0.0678	0.0984
PC aa C36:5	0.2228	0.3044	0.0000	**2.0378E-09**
PC aa C36:6	0.0069	**0.0201**	0.0000	**2.1361E-08**
PC aa C38:0	0.0295	0.0599	0.0000	**3.8333E-06**
PC aa C38:1	0.3429	0.4465	0.0312	**0.0496**
PC aa C38:3	0.5456	0.6435	0.0010	**0.0024**
PC aa C38:4	0.5447	0.6435	0.0008	**0.0019**
PC aa C38:5	0.5372	0.6435	0.0000	**6.3963E-10**
PC aa C38:6	0.0057	**0.0178**	0.0000	**4.9486E-07**
PC aa C40:2	0.0211	**0.0470**	0.0041	**0.0078**
PC aa C40:3	0.0024	**0.0102**	0.0000	**3.8333E-06**
PC aa C40:4	0.7429	0.7886	0.0096	**0.0170**
PC aa C40:5	0.4073	0.5110	0.0000	**2.5656E-08**
PC aa C40:6	0.0968	0.1553	0.0000	**6.3963E-10**
PC aa C42:0	0.0032	**0.0122**	0.0015	**0.0034**
PC aa C42:1	0.0042	**0.0145**	0.0092	**0.0165**
PC aa C42:2	0.0223	**0.0489**	0.0330	0.0512
PC aa C42:4	0.0335	0.0660	0.1412	0.1968

PC aa C42:5	0.0515	0.0923	0.9858	0.9858
PC aa C42:6	0.0378	0.0724	0.0000	**1.0350E-05**
PC ae C30:0	0.0195	**0.0441**	0.0000	**0.0001**
PC ae C30:1	0.5584	0.6531	0.1653	0.2236
PC ae C30:2	0.1009	0.1601	0.0000	**4.5905E-10**
PC ae C32:1	0.0174	**0.0401**	0.0011	**0.0026**
PC ae C32:2	0.0002	**0.0015**	0.0000	**2.1788E-06**
PC ae C34:0	0.6100	0.6923	0.0000	**1.2274E-06**
PC ae C34:1	0.0023	**0.0101**	0.0000	**7.0769E-06**
PC ae C34:2	0.0006	**0.0034**	0.0130	**0.0217**
PC ae C34:3	0.0004	**0.0026**	0.5559	0.6345
PC ae C36:0	0.0002	**0.0019**	0.0010	**0.0024**
PC ae C36:1	0.1679	0.2439	0.0000	**6.3963E-10**
PC ae C36:2	0.0445	0.0829	0.0000	**2.1253E-06**
PC ae C36:3	0.0046	**0.0151**	0.0271	**0.0445**
PC ae C36:4	0.5031	0.6199	0.0297	**0.0479**
PC ae C36:5	0.8898	0.9164	0.0130	**0.0217**
PC ae C38:0	0.0077	**0.0213**	0.0000	**5.6169E-11**
PC ae C38:1	0.7940	0.8364	0.0019	**0.0040**
PC ae C38:2	0.0053	**0.0171**	0.0019	**0.0040**
PC ae C38:3	0.5855	0.6790	0.0000	**6.3963E-10**
PC ae C38:4	0.7370	0.7884	0.0004	**0.0011**
PC ae C38:5	0.8844	0.9164	0.0038	**0.0074**
PC ae C38:6	0.2554	0.3455	0.0000	**2.4905E-06**
PC ae C40:1	0.0662	0.1101	0.0000	**5.1511E-07**
PC ae C40:2	0.6717	0.7416	0.0000	**2.7917E-08**
PC ae C40:3	0.0041	**0.0145**	0.0000	**6.1928E-07**
PC ae C40:4	0.2097	0.2924	0.0127	**0.0217**
PC ae C40:5	0.5415	0.6435	0.0000	**1.6829E-05**
PC ae C40:6	0.1478	0.2193	0.0000	**2.6195E-09**
PC ae C42:1	0.6337	0.7052	0.8511	0.8966
PC ae C42:2	0.1154	0.1772	0.0000	**4.3643E-09**
PC ae C42:3	0.0014	**0.0067**	0.0000	**0.0001**
PC ae C42:4	0.0269	0.0571	0.9562	0.9631
PC ae C42:5	0.0492	0.0893	0.3049	0.3861
PC ae C44:3	0.9836	0.9836	0.0031	**0.0063**
PC ae C44:4	0.0257	0.0555	0.0646	0.0948
PC ae C44:5	0.1072	0.1681	0.6795	0.7442
PC ae C44:6	0.0153	**0.0357**	0.4564	0.5525
**Sphingomyelins**				
SM (OH) C14:1	0.2761	0.3663	0.0000	**6.3963E-10**
SM (OH) C16:1	0.4203	0.5225	0.0000	**6.3963E-10**
SM (OH) C22:1	0.0611	0.1054	0.0000	**5.1371E-09**
SM (OH) C22:2	0.0013	**0.0067**	0.0000	**6.3963E-10**
SM (OH) C24:1	0.9714	0.9785	0.0001	**0.0002**
SM C16:0	0.0005	**0.0029**	0.0000	**2.6293E-06**

SM C16:1	0.0000	**4.6000E-05**	0.0000	**2.3341E-08**
SM C18:0	0.0369	0.0718	0.0000	**3.4305E-07**
SM C18:1	0.0035	**0.0132**	0.0000	**1.1223E-07**
SM C20:2	0.0003	**0.0019**	0.0001	**0.0002**
SM C24:0	0.1470	0.2193	0.0000	**2.3000E-05**
SM C24:1	0.0001	**0.0006**	0.0000	**1.8503E-06**
SM C26:0	0.5321	0.6435	0.0000	**3.8333E-06**
SM C26:1	0.0611	**0.1054**	0.0000	**1.3788E-06**
Hexose (H1)	0.0030	**0.0116**	0.0000	**7.0769E-06**

* P values are from independent t-test.

**FDR-adjusted P values are adjusted for multiple comparisons using the Benjamini–Hochberg procedure.

Note: two age groups; group 1, age <45 years old; group 2, age >=45 years old.

Abbreviations are as follows. Cx:y: x=number of carbons in the fatty acid side chain, y=number of double bonds in the fatty acid side chain; DC: decarboxyl; OH: hydroxyl; lysoPC: lysophosphatidylcholine; PC: phosphatidylcholine; aa: acyl-acyl; ae: acyl-alkyl; SM: sphingomyelin.
